# Robots Learn to Recognize Individuals from Imitative Encounters with People and Avatars

**DOI:** 10.1038/srep19908

**Published:** 2016-02-04

**Authors:** Sofiane Boucenna, David Cohen, Andrew N. Meltzoff, Philippe Gaussier, Mohamed Chetouani

**Affiliations:** 1 Laboratoire ETIS, UCP, UMR CNRS 8051, 95000 Cergy-Pontoise, France; 2 Laboratoire ISIR, Université UPMC, CNRS, 75005 Paris, France; 3 Department of Child and Adolescent Psychiatry, APHP, Groupe Hospitalier Pitié-Salpêtrière, 75013 Paris, France; 4 Institute for Learning & Brain Sciences, University of Washington, Seattle, WA, 98195 USA

## Abstract

Prior to language, human infants are prolific imitators. Developmental science grounds infant imitation in the neural coding of actions, and highlights the use of imitation for learning from and about people. Here, we used computational modeling and a robot implementation to explore the functional value of action imitation. We report 3 experiments using a mutual imitation task between robots, adults, typically developing children, and children with Autism Spectrum Disorder. We show that a particular learning architecture - specifically one combining artificial neural nets for (i) extraction of visual features, (ii) the robot’s motor internal state, (iii) posture recognition, and (iv) novelty detection - is able to learn from an interactive experience involving mutual imitation. This mutual imitation experience allowed the robot to recognize the interactive agent in a subsequent encounter. These experiments using robots as tools for modeling human cognitive development, based on developmental theory, confirm the promise of developmental robotics. Additionally, findings illustrate how person recognition may emerge through imitative experience, intercorporeal mapping, and statistical learning.

By 18 months of age human infants are able to recognize themselves in a mirror. This skill is rare in the animal kingdom, and shared only with a few other mammals (e.g., great apes and elephants)^1^. The ontogenetic factors contributing to this implicit sense of self have been explored^2^. Some component skills are the ability of human infants to discriminate between faces^3^, to compare different inputs and match them across different sensory modalities^4^, and to be sensitive to interpersonal synchrony^5^,^6^.

These findings and others raise the intriguing possibility that young infants may be able to detect and use the equivalences between felt acts of the self and visible acts of the other^7^ prior to language and before they have compared self and other in a mirror. Here, we use computational modeling and robotics to illuminate a key aspect of preverbal social cognition - how infants use social encounters, especially naturally occurring mutual imitation between adult and child, to help recognize individuals when they are reencountered at another point in time. The experiments reported here use a wide range of social agents, including typically developing adults and children as well as children with autism spectrum disorder (ASD), and avatars.

It has been demonstrated that in interpersonal interactions preverbal infants do not just recognize that another moves when they move (temporal contingency), but that another acts in the same manner as they do (structural congruence)^6^,^7^,^8^. This has been shown by measures of increased attention and positive affect at being imitated, as well as by neuroscience measures acquired during mutual imitation episodes (mu rhythm responses in the infant electroencephalogram, EEG)^9^. Such recognitions imply that there is a coding of one’s own body and its relation to the body of others prior to language, and raises the idea that preverbal action imitation is a mechanism for social learning as sought by evolutionary biologists^10^, and a channel for preverbal communication^11^. Within developmental psychology, the recognition of personal identity is thought to be a crucial developmental milestone, because infants need to re-identify a person as “the same one again” after a break in perceptual contact and after changes in appearance (putting on a kerchief, growing a beard, getting a haircut)^12^. Once the child has language, this re-identification is solved through the use of proper names and verbal queries. Prior to language, infants use nonverbal actions as part of their re-identification routines. For infants, it has been shown that re-identification of the same person is not based solely on morphological features of the face, but also includes how that person interacts with the infants and the social games they play together. Many action games derive from mutual imitation encounters, which led Meltzoff and Moore to postulate a “social identity function” for imitation^13^,^14^.

Cognitive developmental robotics links developmental science with robotics using interdisciplinary insights^15^,^16^,^17^ that integrate two complementary directions of thought. On one side, it seeks to deepen our understanding of higher human cognitive functions by using a synthetic approach^16^,^18^. On the other side, it seeks to implement learning mechanisms in robotic platforms to achieve some developmental activities that are at play in promoting children’s cognitive development^17^,^18^,^19^,^20^,^21^. Learning through imitation has been achieved in developmental robotics using specific algorithms and sensory-motor architectures using artificial neural networks (N.N.)^22^,^23^. So far untested is whether imitation learning during robot-human interaction allows the development of another cognitive ability such as the robot’s ability to recognize a human partner that is encountered again at a subsequent time.

Here, inspired by the idea of a social identity function for imitation, we show that a computational learning architecture (Fig. 1a) combining a N.N. for extraction of visual features (*VF*), robot’s motor internal state (*MIS*), motor internal state prediction (*MISP*) that associates *VF* and *MIS* activities allowing posture recognition, short term memory (*STM*), and novelty detection (Fig. 1b) was able to learn through mutual imitation encounters how to recognize a person at a later point in time. From previous work, we know that a similar architecture without the novelty detector was able to learn by imitation^22^,^23^. In addition, the architecture was able to distinguish the social signature of the interactive partners (typically developing children, children with ASD, and adults)^22^ as evidenced by the number of neurons needed to learn from *VF* that significantly increased during learning with children with ASD compared to other partners^22^. Finally, during learning by imitation, the same metrics showed a sudden increase when the robot changed from a single interactive partner to another, whoever the partner^22^,^24^. Thus, we hypothesized that coupling the number of neurons needed to learn from VF with a novelty detector would help in achieving person recognition. To do so, the novelty detector produces feedback, since the robot needs mechanisms of self-assessment for regulating and modulating learning. As a consequence, the robot is able to evaluate its learning. This mechanism, based on prediction error, can be used with sensory-motor coupling and is detailed on Fig. 1b and in the method section. Prediction error provides a measure of learning progress. In our case, we hypothesized that the sensory-motor architecture learns connections between perceptions and actions, and the self-assessment (prediction error) allows the robot to detect a new event (in our experiments, a new partner).

To evaluate robotic learning, we performed three experiments using the same computational learning architecture in different contexts (Fig. 2). Key experiment differences included changing partners (child, adult or avatar), motor imitation tasks (involving arms or face), and robotic platforms. In Experiment 1, the robot Nao, interacted with a human partner - either a typically developing (TD) child, a child with autism spectrum disorder (ASD), or an adult during a motor imitation task (5 postures). After the learning phase, the results show that Nao is able to accurately recognize the interactive agents in a subsequent encounter. In Experiment 2, we changed both the imitation task (5 facial expressions) and robotic platform (Robot head) and obtained a similar transfer for person recognition after the learning phase. In Experiment 3, Nao interacted with a set of avatars having very similar visual characteristics to each other, and “personal identity recognition” (here avatars’ visual motor/motion characteristics) was again achieved.

## Results

Experiment 1 used a motor imitation task (5 postures, see supporting online materials, Figure S1) between a Nao robot and a human partner. Three groups of people interacted with the humanoid robot: 15 typically developing (TD) children, 15 children with autism spectrum disorder (ASD), and 11 adults (participants’ characteristics are available in Table S1). N.N activities during the learning phase are shown in Fig. 3. The number of neurons needed to learn in the Visual Feature N.N. increases as a function of the number of interactive participants (red line). When a new participant is introduced during the learning phase, the novelty module shows an important activity (green line) and a brief synchronous hit occurs in the Person Recognition N.N. corresponding to the recruitment of a specific artificial neuron per participant. To test how the architecture developed person recognition, we presented 100 different images per participant to the system in four conditions using two dichotomous contrasts: (i) images shown randomly or not and (ii) images previously watched during the learning phase or not. In terms of recognition performance rates (Fig. 4a), we found that the architecture was able to recognize on average 68.2% (95% CI:66.7–69.7), 81.1% (95% CI:79.9–82.3), 59.4% (95% CI:58–61), and 67.1% (95% CI:65.6–68.5) of the 41 participants (respective binomial tests: random/known, *p* < 0.001; non-random/known, *p* < 0.001; random/unknown, *p* < 0.001; non-random/unknown, *p* < 0.001). Using a general linear mixed model-GLMM, we found that recognition was significantly better for known pictures (as opposed to unknown; *β* = 1.19, *p* < 0.001) and lower for pictures shown randomly (as opposed to non-randomly, *β* = −0.51, *p* < 0.001). Recognition rate per participants and conditions are shown in Figure S2. Four recognition scores were highly correlated (Fig. 4d). Only 5 (12.2%) participants were poorly recognized by the system (rate < 10 times chance levels = 25%). GLMM investigating recognition scores according to participants’ subgroups (Adults vs. TD children vs. children with ASD) are given in Figure S2 caption. On average, recognitions scores were significantly better for adults vs. children with ASD, and better for children with ASD vs. TD children.

Experiment 2 tested the generalizability of our modeling in another interactive context. We changed the task and the robotic platform by using an expressive robot head in an emotional interaction paradigm. The robot head learned through a motor facial imitation task (4 emotional facial expressions plus a neutral face; see online Figure S3) with 25 adult participants who imitated the facial expressions of the robotic head. N.N activities during the learning phase was similar to those in Experiment 1 (see Supporting Online Material, Figure S4). The architecture was able to recognize on average 94.7% (95% CI:94–95.4), 95.5% (95% CI:94.9–96.2), 77.9% (95% CI:76.7–79.1), and 86% (95% CI:85–87) of the 25 participants (respective binomial tests: random/known, *p* < 0.001; non-random/known, *p* < 0.001; random/unknown, *p* < 0.001; non-random/unknown, *p* < 0.001), see Fig. 4b. Recognition was better for known pictures (as opposed to unknown; GLMM: *β* = 1.46, *p* < 0.001) and lower for pictures shown randomly (as opposed to non-randomly, GLMM: *β* = −0.79, *p* < 0.001). Recognition rate per participant and condition are summarized in supporting online material Figure S5. Only 1 (4%) individual was poorly recognized by the system (rate < 10 times chance levels = 40%).

Because the visual system was based on the sequential exploration of the image focus points and there was no constraint on how the local views were selected, objects in the background and/or irrelevant parts of the human body during learning could have been distracting. Experiment 3 was carried out to control for unexpected visual cues that may have contributed to recognition scores (e.g. the color of a participant tee-shirt) and to show the robustness of visual features learning based on focus points. To achieve this goal, Nao was imitated by a set of 12 avatars that were highly similar in their visual presentation (white humanoids) but with specific traits that we systematically manipulated (length or width of the arms, head or body sizes, see supporting material, Figure S6). The results showed that the N.N activities during the learning phase were similar to those in Experiment 1 (see Figure S7). The architecture was able to recognize on average 84.1% (95% CI:83–85.3), 93.1% (95% CI:92.3–93.9), 84.7% (95% CI:82.7–86.7), and 89.6% (95% CI:87.9–91.3) of the 12 avatars (respective binomial tests: random/known, *p* < 0.001; non-random/known, *p* < 0.001; random/unknown, *p* < 0.001; non-random/unknown, *p* < 0.001), see Fig. 4c. Recognition was better for known pictures (as opposed to unknown; GLMM: *β* = 0.75, *p* < 0.001) and lower for pictures shown randomly (as opposed to non-randomly, GLMM: *β* = −0.98, *p* < 0.001). Recognition rate per participant and condition are summarized in supporting material Figure S8. Only 2 (16%) avatars were poorly recognized by the system (rate < 10 times chance levels = 83%).

## Discussion

In all three experiments we used developmental robotics and computer modeling to implement a test of the idea that preverbal mutual imitation of actions between infant and caretaker may support a social identity function. Based on prior human infant work^13^,^14^, we predicted that imitative experiences during robot-human interaction would enable the robots to recognize a human partner that the robot had already encountered. The results accord with this prediction. During mutual imitation episodes, the robot learns signature actions, postures, and facial expressions, and an emergent property obtains (person recognition). Our model used a sensory-motor architecture based on neural networks (Hebb conditioning)^22^,^23^,^24^ coupled with an auto-evaluation mechanism based on prediction error to detect a new social partner (perceptual novelty).

Five points are relevant to theories of robotic and human learning and development. First, the architecture enabled the simultaneous development of perceptual, motor, and cognitive abilities. This coupling of perceptual-motor and cognitive development was originally highlighted as a key aspect of cognitive development by Piaget^25^ and Wallon^26^ and is now incorporated in the field called “action science”, encompassing modern neuroscience, computer science, cognitive science, and developmental psychology, which was capitalized on here^27^.

Second, the current work fits with the idea that the representation of the body is important to social-cognitive development. This view has roots in the philosophical insights of Merleau-Ponty^28^ and has been taken up by theorists who use the term “embodiment”^29^,^30^. To bring greater precision to this general idea, we used robots because we could implement our model in a rigorous way by strictly controlling and specifying the behavior of robots. From our point of view, a different body involves a different learning. We hope that research such as this will help scientists to integrate significant aspects of “embodiment,” sensory-motor, and cognitive-developmental approaches. The fundamental fact is that infants (and robots) do have bodies and these play a significant role in their initial social learning and development, as illustrated in the current experiments and recent infant neuroscience theory and data^31^. Indeed, our model needs a sensory-motor internal state to proceed with imitation learning. The robot is able to learn because it acts in the environment and with others. The robot connects what it sees with what it does (corresponding to a perception-action mapping). In addition, the results show that a mirroring mechanism, the sensory-motor architecture, and a self-evaluation mechanism (error prediction and evaluation) are sufficient to develop an autonomous robot in which imitation is an important element in the interaction and allows the learning of a complex ability such as person recognition. Previous work on interpersonal interaction has highlighted the centrality of motor dynamic similarity in joint action^32^ and in human robot interaction^33^. Other studies in robotics^34^,^35^,^36^ have discussed the role of learning and social referencing. However, the current work goes further by proposing a developmental approach in which a real self-supervised developmental sequence can emerge. We designed a model allowing learning through interaction by using low level features and minimal knowledge to avoid the symbol grounding problem (the problem of how symbols get their meanings)^37^.

Third, the child-robot interactions involved imitative encounters. Developmental scientists working with human infants^7^,^11^,^38^, have pointed out that mutual imitation games are a common occurrence among infants and caretakers, and provide a rich learning experience not only because they include structural matches, but also because they include a temporal component. Interpersonal temporal synchrony of the type occurring in mutual imitation is now considered as a social signal per se^5^,^6^ and has been associated with both neural^39^ and hormonal changes in humans^40^,^41^. In the current experiments, the robot-human interaction was treated as a single global system where the robot could learn based on the interaction - hence, the robot-human system was considered an autopoietic social system that was sufficient to maintain the interaction and to develop and regulate the robot’s behavior. The robot would not have developed without the help of the human agent (caregiver). Our computational modeling served to render this kind of social learning more precise and quantifiable than is often the case within developmental and clinical psychology.

Fourth, consistent with work showing that infants react to being imitated in special behavioral and affective ways, exhibiting a distinctive infant neural response to having their behavior copied (reduction in mu rhythm in the EEG)^42^, the human partner in the current robot experiments was considered as a mirror during the learning phase. Consequently, the robot could learn to connect what it saw with what it did. When the sensory-motor architecture was used in this learning context and coupled with the novelty detector, this was sufficient to develop new autonomous behaviors (person recognition).

Finally, it is of note that the novelty detector computational architecture was based on statistical/probabilistic learning that has been shown to be a key mechanism during early language acquisition^43^,^44^ and that has been recently used in robotic goal-based imitation learning^45^. The novelty detector system that we used made use of a special ‘prediction error’ calculation that helped isolate input from the environment that was unexpected and therefore important to learn about. Our current model and experiments involved typically developing children in child-robot interaction, children with ASD in child-robot interaction, as well as adult-robot interaction, and avatar-robot interaction. This suggests that the architecture allows for generalized learning across a broad range of agents and interactive participants.

In summary, the experiments illustrate that (i) robots learn to recognize individuals from imitating adults, children with autism, and other agents; (ii) robots can be used as tools for modeling cognitive development, based on developmental theory, confirming the promise of developmental robotics; (iii) in our computational model, person recognition spontaneously emerges through imitation learning, intercorporeal mapping, and statistical learning.

## Method

### Participants

Participants characteristics involved in experiment 1 and 2 are given in supplementary Table S1. Patients with ASD were followed in a specialized clinic of the Pitié-Salpétriêre hospital. Typically developing (TD) children were recruited from several schools in the Paris area. They were matched to the children with ASD with respect to their developmental ages and genders. Children with ASD were assessed with the Autism Diagnostic Interview-Revised (ADI-R) to assess ASD symptoms. The psychiatric assessments and parental interviews were conducted by two child psychiatrist/psychologists who are specialized in autism. The developmental age was assessed using a standardized cognitive assessment. In these experiments, we used a group of TD children to address whether age and/or morphology could affect the recognition results of the architecture. Also, we included a group of children with ASD to address whether peculiarities in social interaction that are impaired in children with ASD^46^ would affect the recognition results. Adults participating in experiment 1 and 2 were University students from Medical and Engineering schools. Each participant has performed the experiment with the robot only one time. The protocol was approved by the Pitié-Salpétriêre hospital ethics committee (Comité de Protection des Personnes). All the parents or participants received written and oral information on the experiment and gave written consent before their participation or the participation of their child. All experiments were performed in accordance with relevant guidelines and regulations.

### Experimental procedure

We adopted a developmental approach whereby a robot learned through interaction with a partner. Posture recognition was learned autonomously using a sensory-motor architecture through an imitation game between the partner and the robot. Figure 2 (middle column) shows an overview of the experimental design for all the experiments. First, we aimed to investigate how a robot could learn to properly imitate a person’s posture during an interaction composed of two phases. During the learning phase, the robot produced a random posture selected from a defined set of postures, and the participant imitated the robot; then, the robot mapped what it did to what it saw. The architecture enables learning without explicit teaching signals (see details below). This first phase lasted 3 min and learning occurred in less than 2 min, after which the roles were reversed. During the validation phase, the robot then had to imitate the posture of the partner who now was leading the imitation interaction. During the first phase, the robot learns the task, but also records all the images. Consequently, a database is created to perform offline processing. Each image was annotated with the response of the robot during the online learning. All the images are correctly labeled because the participant mimics the robot’s postures. After the mutual imitation encounter, we presented for a second time each partner through a set of pictures for testing recognition (see below). To show the generalizability of our sensory-motor approach when the robot is immersed in different learning contexts, three different experiments were carried out (Fig. 2) by changing either populations (e.g. children with ASD or avatars), robotic platforms (e.g. complete robot or Robot head), or motor abilities (e.g. facial motor expression or body posture). Regarding robotic platforms, we selected Nao because it has been used in several studies on Robot/Human interaction with individuals with ASD^47^ and has shown excellent performance and acceptance by the children with ASD^22^,^48^. Since Nao’s motor abilities are mainly located on arms and body posture, we selected the robot head for experiment 2 to offer a novel motor activity.

### Sensory-motor architecture: PerAc architecture

Here, we describe the sensory-motor architecture that enables the learning, recognition and imitation^22^,^23^. We also summarize the properties of the generic sensory-motor architecture (PerAc architecture) used as a building block in our model. PerAc learns sensory-motor conditionings in order to form a perception as a dynamical sensory-motor attractor. It involves two data streams associated respectively to perception and action. From each perceived input, we can extract reflex information to control directly the robot action: the low-level pathway consists of reflex behaviors. We also add a mechanism for recognizing the sensory input patterns that can take control of the robot’s actions and avoid the reflex pathway: the conditioning pathway allows anticipating reflex behaviors through the learning. When new PerAc associations have been learned, they appear like a new reflex pathway that can support a new level of association (a recursive mechanism). This learning performs associations between the recognition of sensory information (high-level) and the reflex behavior (low-level). Learned links can, thus, be considered as meta-reflexes.

**Figure 1:**
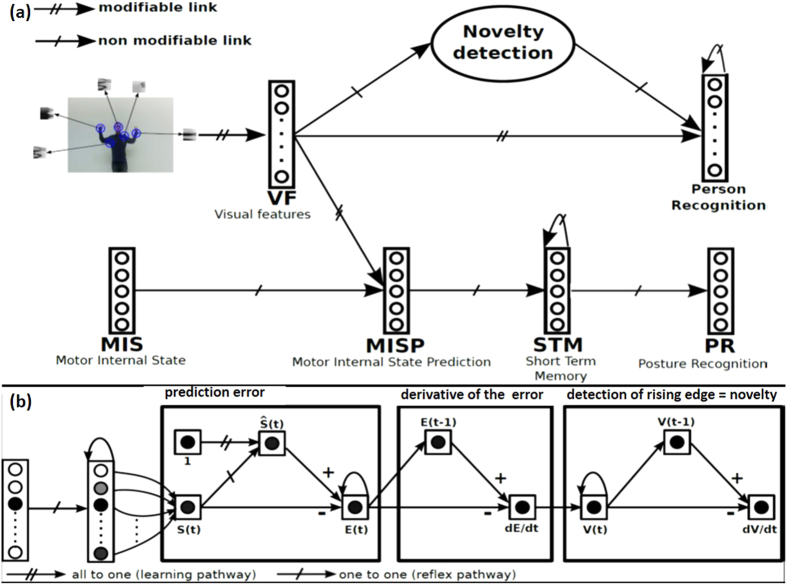
Sensory motor architecture allowing the learning of personal identity through motor imitation **(a)** (PR = Posture Recognition). The novelty detection coupling (**b**) allows the robot to detect novelty in the visual sensations. Three principles are used: (i) prediction error; (ii) derivative of error; (iii) detection of rising edge (novelty). The robot learns the sensory-motor contingency of a given strategy by learning to predict the current sensation from the previous perception.

**Figure 2:**
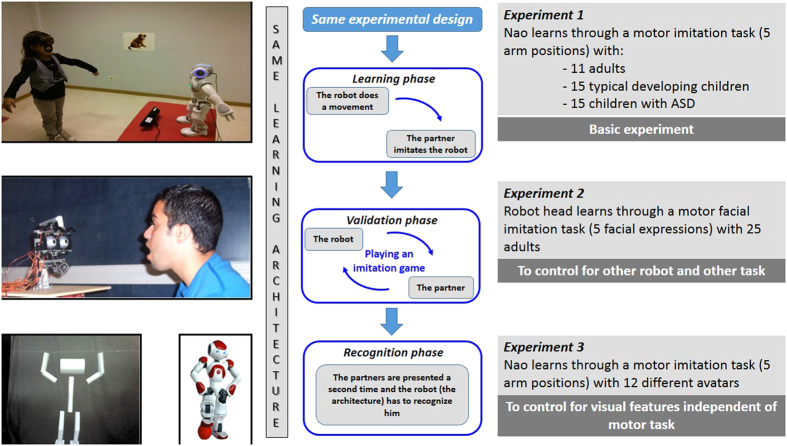
Overview of the experiments showing imitation learning and partner’s recognition during interaction between a robot and a partner. The current experiments used the same learning architecture and varied the learning context: (Experiment 1, top) posture imitation between the robot Nao and human partners; (Experiment 2, middle) facial imitation between Robot Head and human partners; (Experiment 3, lower) posture imitation between the robot Nao and avatars partners.

**Figure 3:**
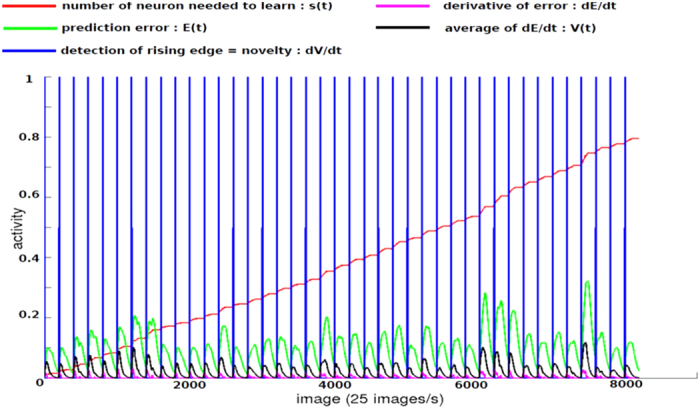
Neural network (N.N.) activities during the learning phase in experiment 1. The number of neurons needed to learn from the Visual Features N.N. (red); Prediction Error E(t) from the novelty detector N.N. (green); derivative of error prediction *dE*/*dt* (dark pink); average of *dE*/*dt* (black); novelty detection in the Person Recognition N.N. (dark blue); each hit corresponds to the detection of a novel interactive partner.

**Figure 4:**
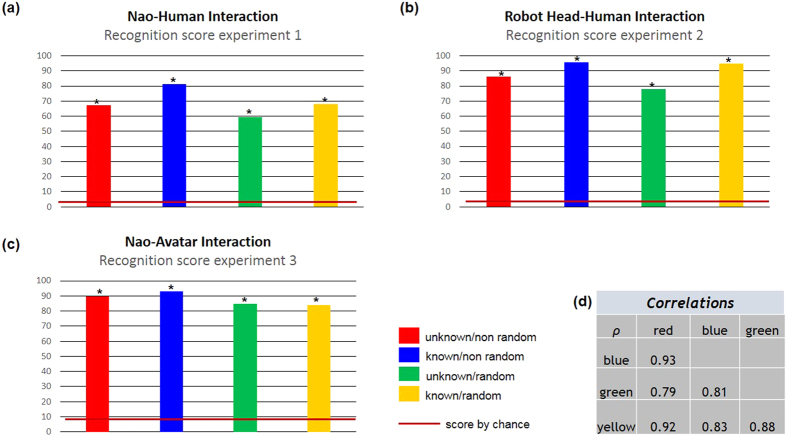
Main results. Mean recognition scores from Experiment 1 (**a**), Experiment 2 (**b**), Experiment 3 (**c**) in unknown/non random (red); known/non random (blue); unknown/random (green); known/random (yellow) conditions. *p < 0.001, comparisons between mean recognition scores and chance were provided by binomial test. (**d**) Correlation matrix between recognition scores from Experiment 1 are shown in the table (red indicates unknown/non random condition and so on).

Within the architecture, visual processing enables the extraction of local views (Fig. 1a). Each local view is then learned by the *VF* (visual features) neural network (N.N.) The *MISP* (motor internal state prediction) N.N. learns the association between the visual features and *MIS* (motor internal state). The robot interacts with the human partner to learn autonomously the posture recognition. The partner is considered as a mirror: the robot produces its first posture according to its internal state (*MIS*) and the human partner then imitates the robot’s posture, thereby enabling the robot to link these postures with its internal state^22^,^23^. Conventional HRI (Human Robot Interaction) architectures are exploiting a multi-stage detection/recognition pipeline: person localization then, posture recognition is performed on the person. In this case, the quality of the results is dependent on the accuracy of the person localization^24^. Consequently, the overall performance, here the generalization capability of the neural network can be affected. From an autonomous learning perspective, it is useful to avoid an ad hoc framing mechanism. Our solution consisted of eliminating the framing step and directly using all the local views around the most activated focus points in the image. Four steps are described below: the visual processing system (or focus point detection), the visual extraction system, the classification approach to learn postures, and the novelty detector.

### The visual processing system: Focus points detection

The visual system was based on the sequential exploration of the image focus points. There was no constraint on how the local views were selected. This means that distractors can be taken on objects in the background and/or irrelevant parts of the human body. The visual attention on potentially interesting regions was controlled by a reflex mechanism allowing the robot to focus its gaze. The focus points were the result of a local competition performed on the convolution between a DOG (difference of Gaussians) filter and the norm of the gradient of the input image. This process allowed the system to focus more on the corners and ends of the lines in the image. For each focus point in the image, a local view centered on the focus point is extracted: a log-polar transform was applied to obtain an input image or a vector more robust to the rotations and distance variations.

### The visual extraction system : Visual features (*VF*)

Figure 1a shows the sensory-motor architecture that enabled the learning, recognition and imitation of postures. The extracted local view around each focus point was learned and recognized by a group of artificial neurons *VF* (visual features) allowing online learning and in real time:


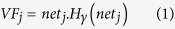



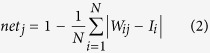


*VF*_*j*_ is the activity of neuron *j* in the group *VF*. *H*_*γ*_(*x*) is the Heaviside function:


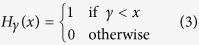


Here, *γ* is a vigilance parameter (the threshold of recognition). When the prototype recognition is below *γ*, then a new neuron is recruited (incremental learning).

This model enables the recruitment to adapt to the dynamics of the input. Thus, *γ* can be set to a low value to maintain a minimum recruitment rate. The learning rule allows both one-shot learning and long-term averaging. The modification of the weights (*W*_*ij*_) is computed as follows:


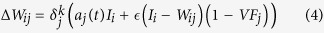


with *k* = *ArgMax*(*a*_*j*_), *a*_*j*_(*t*) = 1 only when a new neuron is recruited; otherwise, *a*_*j*_(*t*) = 0. Here, 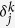 is the Kronecker symbol:


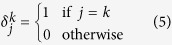


The adaptation rate *ε* performs long-term averaging of the stored prototypes. When a new neuron is recruited, the weights are modified to match the input (the term *a*_*j*_(*t*)*I*_*i*_). The other part of the learning rule, *ε*(*I*_*i*_ − *W*_*ij*_)(1 − *VF*_*j*_), averages the already learned prototypes (if the neuron was previously recruited). The closer the inputs are to the weights, the less the weights are modified. Conversely, the further the inputs are from the weights, the more the weights are averaged. The quality of the results depends on the *ε* value. If *ε* is chosen to be too small, it only has a small impact. Conversely, if *ε* is too large, the previously learned prototypes can be unlearned. This learning rule enables the neurons in the *VF* group to learn to average the prototypes of postural features (such as an arm).

### The classification approach to learn postures

For the classification approach, only the local views for the person (Fig. 1a) that were correlated with a given robot posture were reinforced. The Widrow and Hoff rule^49^ can be used to learn the image correctly if sufficient focus points can be found on the person during the period over which one image is explored. In our network, motor internal state prediction *MISP* associates the activity of the visual features *VF* with the current motor internal state *MIS* of the robot, a simple conditioning mechanism is used, the least mean square (*LMS*) rule. The modification of the weights (*w*_*ij*_) is computed as follows:


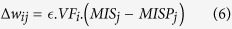


The short term memory (*STM*) is used to sum and filter over a short period. The robot posture is controlled via the *PR* group (a WTA mechanism).

### Novelty detector and prediction error

Novelty detection aims at identifying new or unknown data which differ from the normal data that the architecture was trained with. This process is performed by the identification of outliers in the stream of VF. This detection is motivated by our previous work on the dynamics of the number of neurons needed to learn from VF that unveiled a group signature (typical developing children/adult vs. ASD)^22^. The novelty detection is designed to distinguish the difference between prediction error caused by the model insufficiency and prediction error by novelty in the VF. Thanks to this novelty detector, the architecture has a direct feedback on the quality of the learning allowing robot’s self-evaluation. The self-evaluation mechanism allows the robot to regulate its learning by differentiating the sensory-motor associations which are known, unpredicted by the current system, or in progress^50^. The novelty detection produces a rich signal that enables and triggers the learning of specific events.

The novelty detection is computed from analysis of the prediction error, where changes may indicate some form of novelty. This mechanism has been implemented as follows. The error *e*(*t*) is computed as the difference between the predicted signal 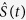 and the actual signal *s*(*t*):


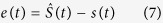


We exploit the mean error *E*(*t*):


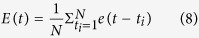


The learning process is proportional to the negative gradient of the mean error:


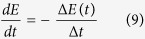


The final step of the novelty detector is the computation of *dV*/*dt* (Fig. 1b) and more precisely rising edges of this signal. The rising edge is interpreted as the detection of a new event (a new person interacting with the robot). In our model, the novelty detector is used to trigger the learning of new person postures.

### Testing person recognition

During learning by imitation, the architecture recruits for each participant an artificial neuron in the person recognition N.N. As a consequence, the number of recruited neurons is equal to the number of participants N in a given experiment. If person recognition is an emerging property acquired during learning by imitation and stable after the learning phase, then the architecture should converge towards activation of the corresponding participant’s artificial neuron among the N neurons available when the participant is presented to the architecture again. During the learning phase (less than 2 min), the robot sees a participant imitating the robot’s movements. This interaction is seen as a video sequence of thousands of images. For computational needs the model only selects 1/10 images through the video sequences. As a consequence, the video sequences could be divided into a set of pictures already known during the 2 min learning phase, and a set of pictures never watched during the learning phase (9/10 images) or during the last minute of the experiment since learning in all cases lasted less than 2 min. To test how the architecture developed person recognition, we used the following protocol with 100 images (for each condition) per participant presented consecutively in 4 conditions using two contrasts: images shown randomly or not; images previously watched during learning phase or not. Here, random refers to static images from a video sequence being shown in a random order. Therefore the 4 conditions were as follow: (i) images were shown randomly among images already watched during learning by imitation (corresponding to testing condition random/known); (ii) images were shown in the same order and among images already watched during learning by imitation (corresponding to testing condition non random/known); (iii) images were shown randomly among images never watched during learning by imitation (corresponding to testing condition random/unknown); (iv) images were shown among images never watched during learning but in the same order (corresponding to testing condition non random/unknown). Images never watched during learning were randomly selected in the 3 min duration of the experiment, meaning that at least one third of the images were not correlated to the set of images watched during learning although they may had some resemblance since they correspond to the same subject doing a set of a limited number of movements. This leads to 4 recognition scores per participant (one per condition) and 4 mean recognition scores per experiment (one per condition). Given the 100 image occurrences per participant and condition, the recognition score is a percentage to be compared to response by chance that is equal to (100/N). Given our learning architecture, we expected recognition scores under known conditions to be better than recognition scores under unknown conditions. Also, we expected recognition scores under non random conditions to be better than recognition scores under random conditions as in the latter the system could not rely on *STM* N.N.

### Statistical analysis

Using Matlab, standard statistics were calculated for each recognition score (mean, standard deviation, range, minimum and maximum) for each experiment. Statistical analyses were performed using R Software, Version 2.12.2. To assess whether recognition scores were above chance level or not we calculated 95% confidence intervals (95% CI) using bootstrapping (10000 replications) and p (5%) values using binomial tests. We also calculated the number of subjects recognized per experiment with ratings superior than 10 times the response by chance. To provide detailed assessment of whether the obtained recognition score was moderated by one of the two contrasts of the testing conditions [known pictures (as opposed to unknown) and pictures shown randomly (as opposed to non-randomly)], we used a general linear mixed model-GLMM with the recognition score as the variable to be explained.

## Additional Information

**How to cite this article**: Boucenna, S. *et al*. Robots Learn to Recognize Individuals from Imitative Encounters with People and Avatars. *Sci. Rep*. **6**, 19908; doi: 10.1038/srep19908 (2016).

## Supplementary Material

Supplementary Information
