# Supplementary material for: Robots Learn to Recognize Individuals from Imitative Encounters with People and Avatars
Source: Sci Rep. 2016 Feb 4;6:19908. doi: 10.1038/srep19908 (PMC4741103; doi:10.1038/srep19908)
Supplement: Supplementary Information [file srep19908-s1.pdf]

Supplemental Material

Robots Learn to Recognize Individuals from Imitative Encounters with People and Avatars

Sofiane Boucenna [1], David Cohen [2,3], Andrew Meltzoff [4], Philippe Gaussier [1], Mohamed Chetouani [2]

[1] Laboratoire ETIS, UCP, UMR CNRS 8051, 95000 Cergy-Pontoise, France
[2] Laboratoire ISIR, Université UPMC,CNRS, 75005 Paris, France
[3] Department of Child and Adolescent Psychiatry, APHP,
Groupe Hospitalier Pitié-Salpêtrière, 75013 Paris, France
[4] Institute for Learning & Brain Sciences, University of Washington,
Seattle, WA, 98195 USA

**Table S1. Socio-demographic and characteristics of the participants**			
**Participants in Experiment 1**			
	ASD (N=15)	Typically developing (N=15)	Adults (N=11)
Age, mean *(± SD)*, year	9.25 *(± 1.82)*	8.06 *(± 2.49)*	25 *(± 3.49)*
Male – Female	13– 5	9 – 6	7 – 4
ADI-R, current, mean (± SD)			
Social impairment score	10.77 *(± 5.3)*	Not relevant	Not relevant
Verbal communication score	7.72 *(± 4.22)*		
Non verbal communication score	4.3 *(± 3.5)*		
Repetitive interest score	2.5 (± 1.88)		
Developmental score	3.3 *(± 1.5)*		
Total score	31.1 *(± 5.46)*		
ADI-R, 4-5 years, mean (± SD)			
Social impairment score	17.33 *(± 8.47)*	Not relevant	Not relevant
Communication verb score	13.75 *(± 5.72)*		
Communication non-verb score	8.08 *(± 4.4)*		
Repetitive interest score	5.25 *(± 3.52)*		
Developmental score	3.83 *(± 1.47)*		
Total score	48.25 *(± 7.34)*		
Developmental age, mean *(± SD)*	7.47 *(± 2.9)*	8.06 *(± 2.49)*	25 *(± 3.49)*
IQ*, mean *(± SD)*	73 *(± 14)*	> 80	> 80
GAF score, mean *(± SD)*	40.27 *(± 9.44)*	> 90	> 90
**Participants in Experiment 2**			
	Adults (N=25)		
Age, mean (± SD), year	24.1 *(± 2.87)*		
Male – Female	15 – 10		

ASD=Autism Spectrum Disorder; SD=Standard Deviation; ADI-R=Autism Diagnostic Interview-Revised; GAF=Global Assessment Functioning

*IQ assessed with the Vineland Developmental Score, the PsychoEducational Profile-Revised, the Kaufman Assessment Battery for Children or the Wechsler Intelligence Scale for Children.

Supplementary Material Experiment 1

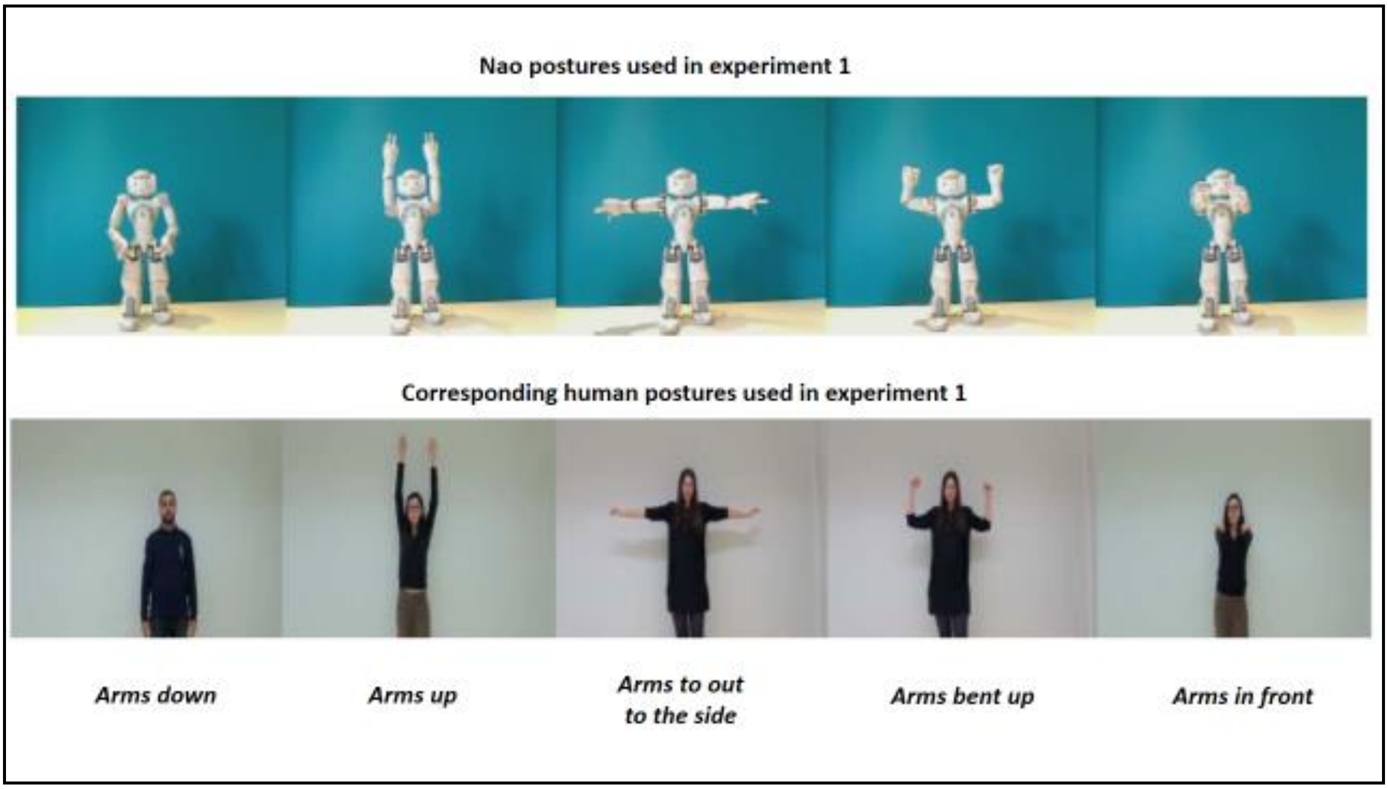

Figure S1. Nao and human postures during the motor imitation task of Experiment 1

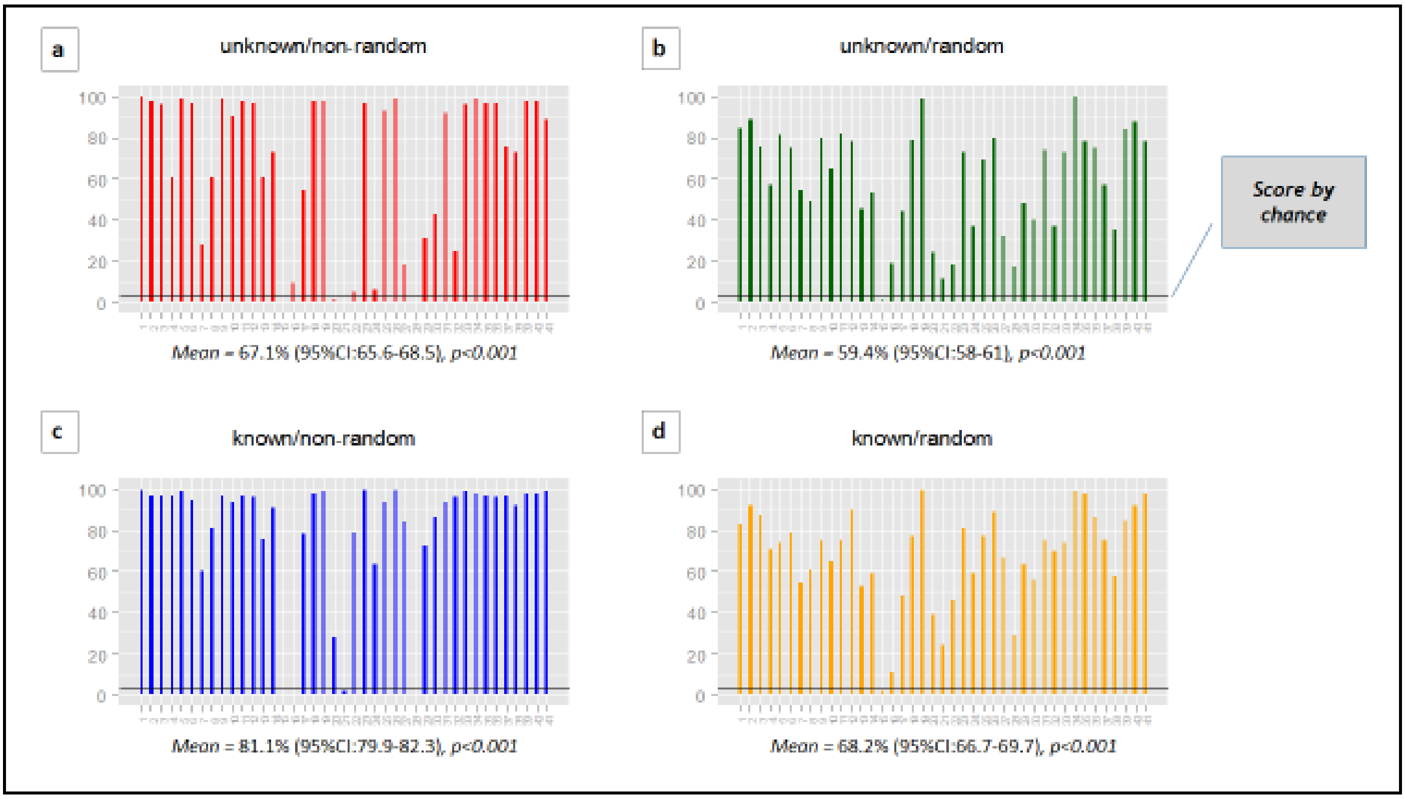

Figure S2. Experiment 1 recognition scores for each of the 41 participants (11 adults, 15 typically developing children and 15 children with Autism Spectrum Disorder) in each of the four conditions: (a) unknown/non-random; (b) unknown/random; (c) known /non-random; (d) known/random. The black horizontal line indicate score by chance level. The missing vertical bars indicate 0.

Using general linear mixed models-GLMM, we found that recognition in unknown / non-random condition was significantly better for adults as opposed to children with ASD (beta=0.88, p<0.001) and as opposed to TD children (beta=1.56, p<0.001), and significantly better for children with ASD as opposed to TD children (beta=0.68, p<0.001). We found that recognition in unknown / random condition was significantly better for adults as opposed to children with ASD (beta=0.5, p<0.001) and as opposed to TD children (beta=1.0, p<0.001), and significantly better for children with ASD as opposed to TD children (beta=0.5, p<0.001). We found that recognition in known / non-random condition was significantly better for adults as opposed to children with ASD (beta=0.56, p<0.001) and as opposed to TD children (beta=1.76, p<0.001), and significantly better for children with ASD compared to TD children (beta=1.2, p<0.001). Finally, we found that recognition in known / random condition was similar for adults as opposed to children with ASD (beta=0.04, p=0.62) and significantly better for adults as opposed to TD children (beta=0.77, p<0.001), and significantly better for children with ASD as opposed to TD children (beta=0.82, p<0.001).

Supplementary Material Experiment 2

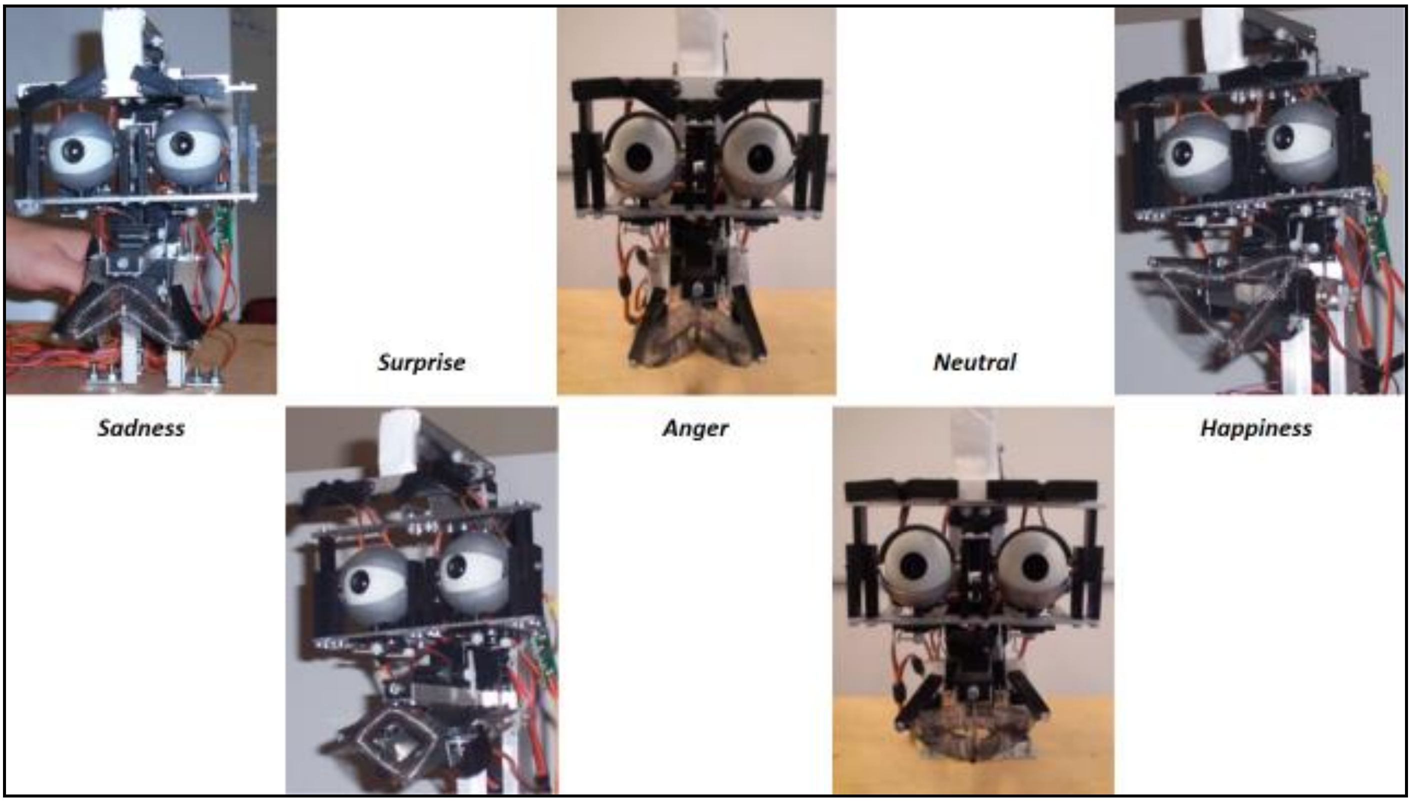

Figure S3. Robot head facial expression during the imitation task of experiment 2

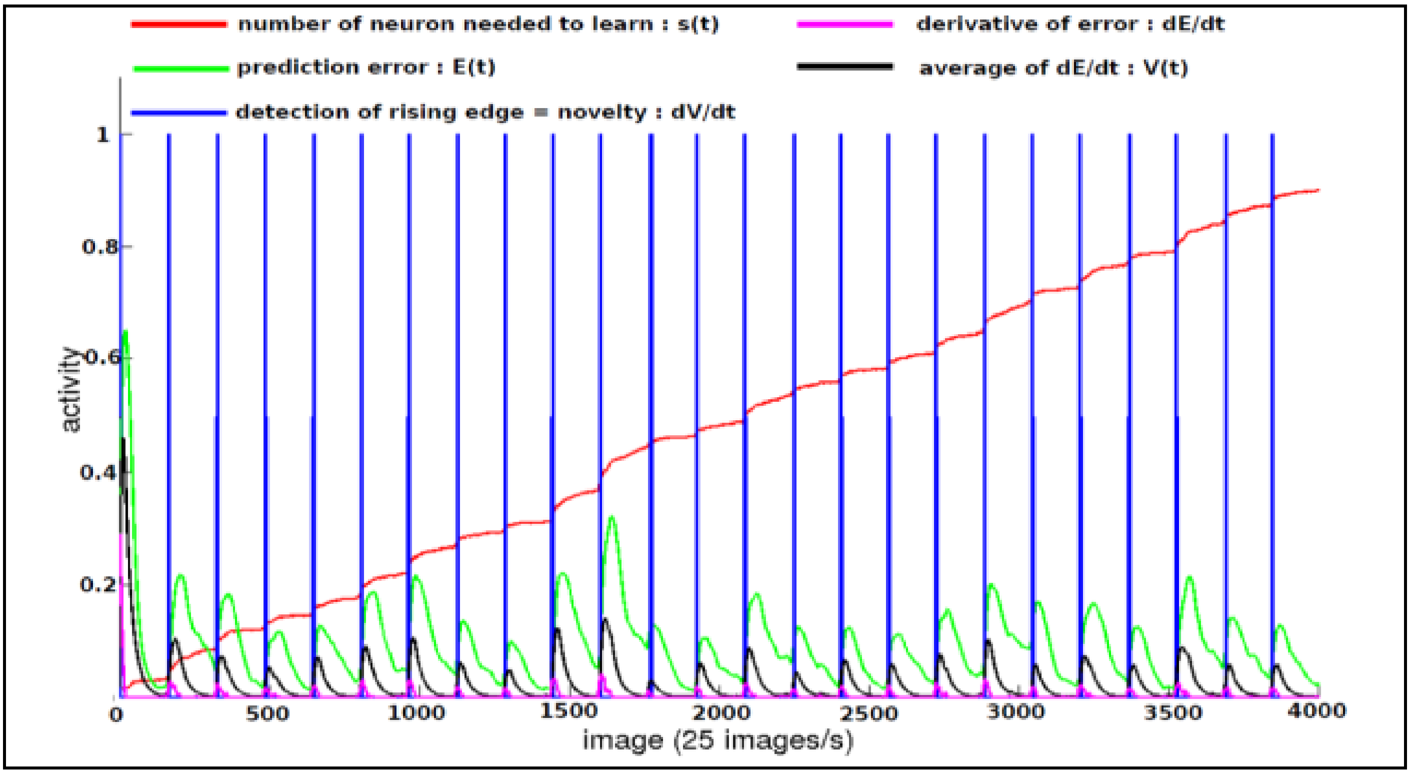

Figure S4. Neural network (N.N.) activities during the learning phase in Experiment 2. *In red: the number of neuron needed to learn from the Visual Features Neural Network. In green: Prediction error E(t) from the Novelty detector N.N. In dark pink: derivative of prediction error dE/dt. In black: average of dE/dt. In dark blue: novelty detection in the person recognition N.N. Each hit corresponds to the detection of a novel interactive partner.*

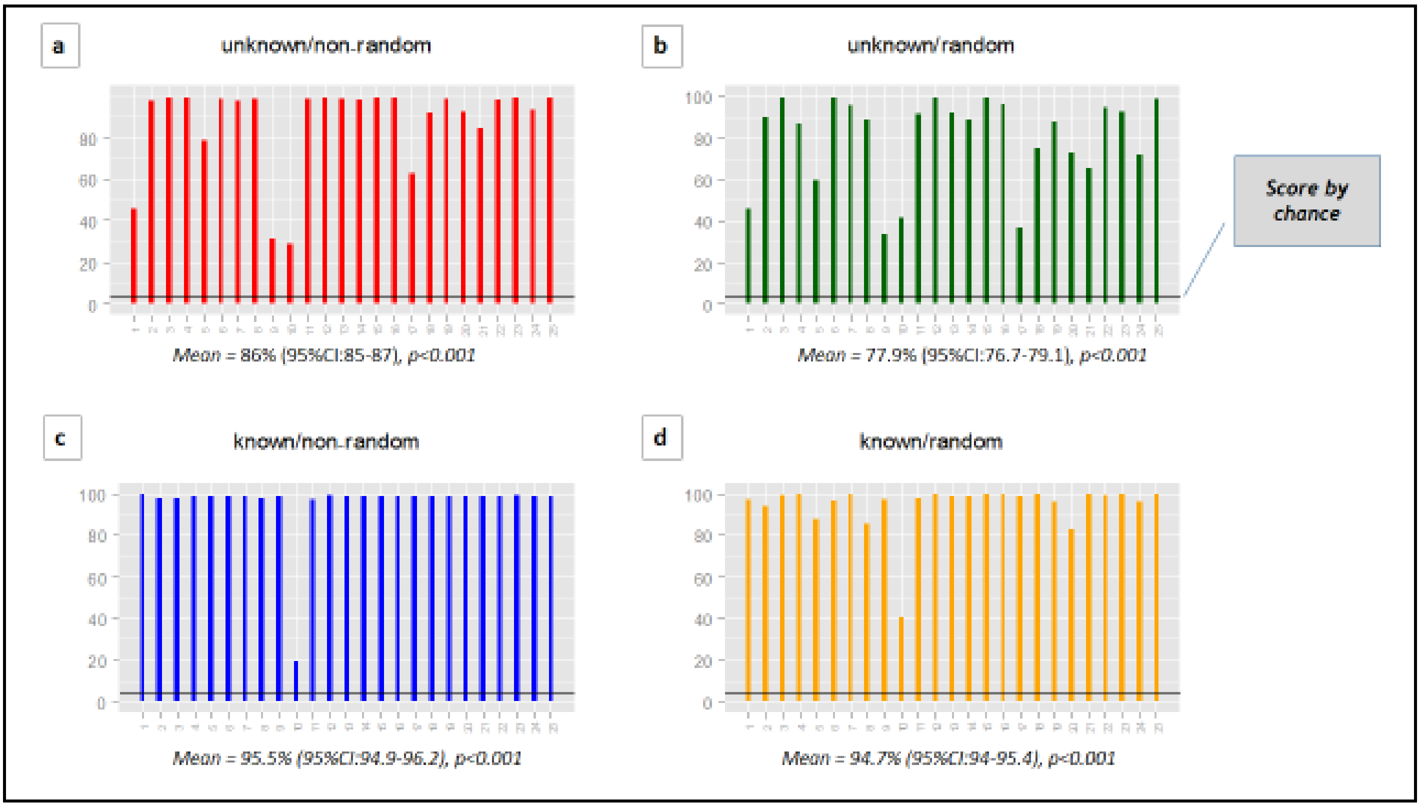

Figure S5. Experiment 2 recognition scores for each of the 25 participants in each of the four conditions: (a) unknown/non-random; (b) unknown/random; (c) known /non-random; (d) known/random. The black horizontal line indicate score by chance level.

Supplementary Material Experiment 3

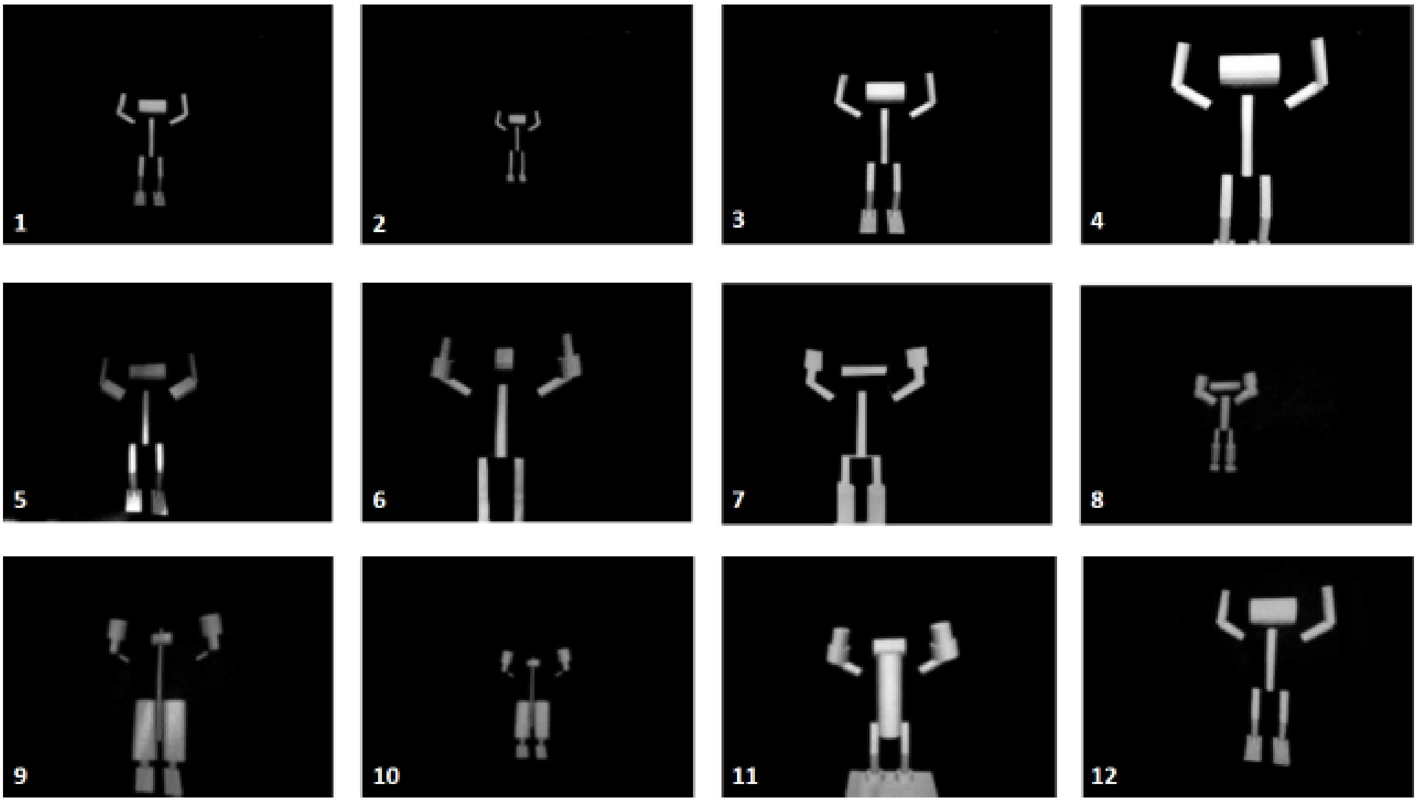

Figure S6. Experiment 3 pictures of the 12 humanoid avatars in the "arms bent up" posture during the motor imitation task with the Robot Nao

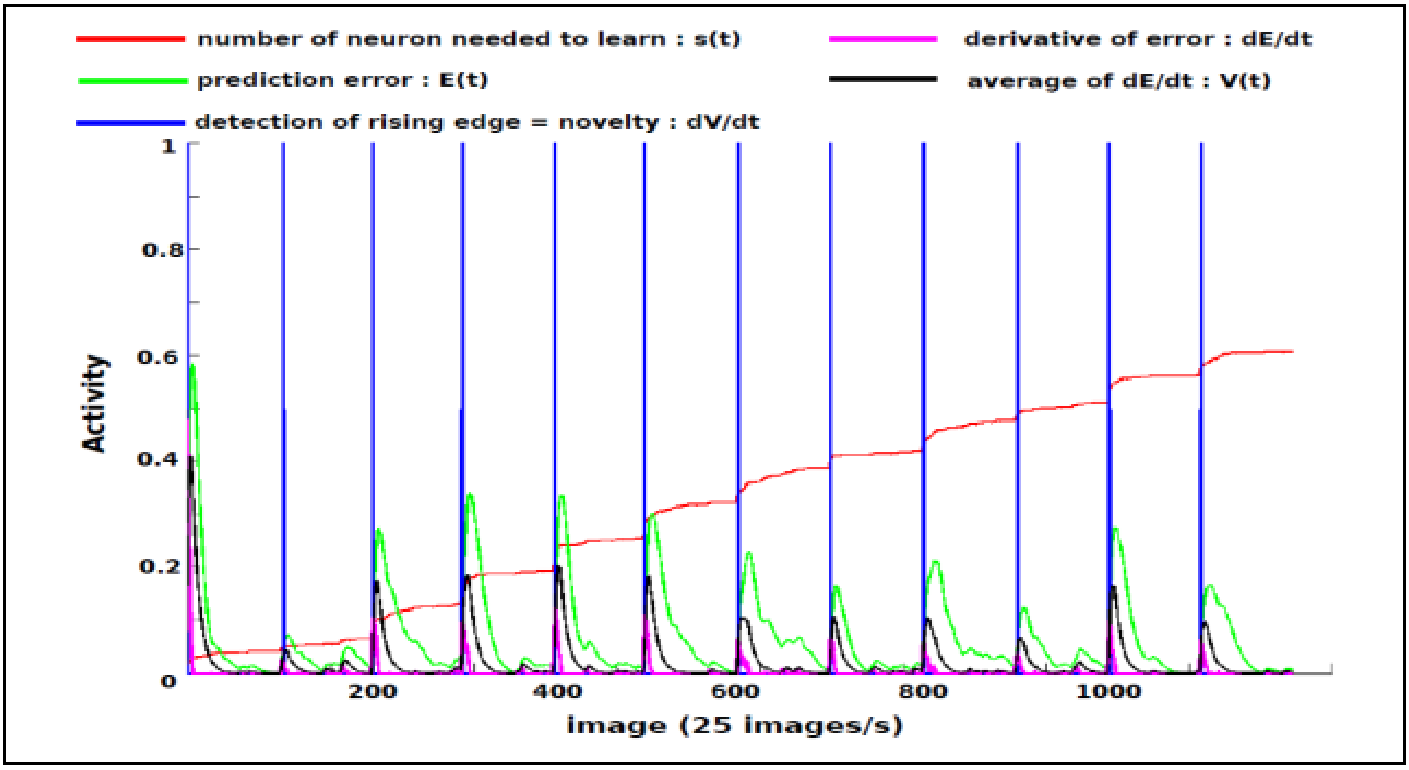

Figure S7. Neural network (N.N.) activities during the learning phase in Experiment 3. *In red: the number of neuron needed to learn from the Visual Features Neural Network. In green: Prediction error E(t) from the Novelty detector N.N. In dark pink: derivative of prediction error dE/dt. In black: average of dE/dt. In dark blue: novelty detection in the person recognition N.N. Each hit corresponds to the detection of a novel interactive partner.*

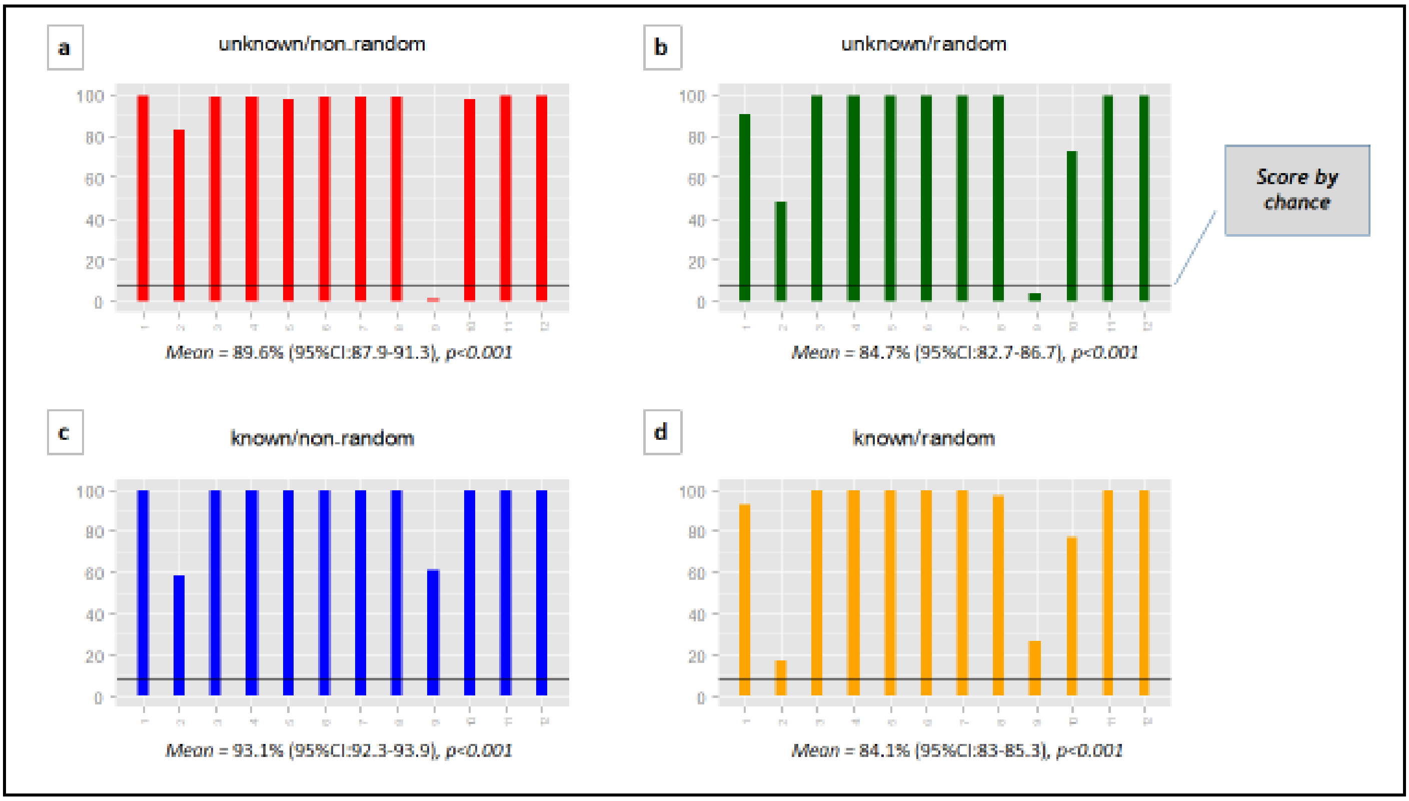

Figure S8. Experiment 3 recognition scores for each of the 12 avatars in each of the four conditions: (a) unknown/non-random; (b) unknown/random; (c) known /non-random; (d) known/random. The black horizontal line indicate score by chance level.
